# Global Perspectives in Acute Kidney Injury: Egypt

**DOI:** 10.34067/KID.0000000000000418

**Published:** 2024-03-21

**Authors:** Mohamed E. Elrggal, Rasha Samir Shemies, Mohamed Hassanein

**Affiliations:** 1 Nephrology Department, AlQabbary Hospital, Alexandria, Egypt; 2 Nephrology Department, Kidney and Urology Center, Alexandria, Egypt; 3 Mansoura Nephrology and Dialysis Unit, Mansoura University, Mansoura, Egypt; 4 Division of Nephrology and Hypertension, University of Mississippi Medical Center, Jackson, Mississippi

**Keywords:** AKI

## Introduction

AKI is a global health problem that affects millions of individuals worldwide and presents a considerable burden on health care systems. In this article, we review the global perspective of AKI in Egypt.

## The Burden and Risk Factors of AKI in Egypt

Egypt is the most populous country in the Middle East and North Africa with more than 102.3 million residents. The prevalence and etiology of AKI in Egypt may differ from those in other parts of the world because of various factors, including low socioeconomic conditions, environmental factors such as pollution, deficient health care infrastructure, lack of health education, and overuse of nephrotoxic drugs such as nonsteroidal anti-inflammatory drugs (NSAIDs), antibiotics, and herbal substances.^1^ The overuse of NSAIDs, antibiotics, and herbal substances is mainly due to the availability of all these medications over the counter, which increases access to these medications by the general population. The risk factors of community-acquired AKI in Egypt include old age, smoking, sepsis, lower education level, and high burden of comorbidities.^2^ In patients with severe trauma, rhabdomyolysis, sepsis, shock, and nephrotoxic medication use have been reported as risk factors of AKI.^3^

## Incidence and Causes of AKI in Egypt

Nationwide data about the incidence and prevalence of AKI in Egypt is lacking. A multicenter study of 500 patients admitted to the intensive care unit (ICU) reported an AKI incidence of 39.7% at admission with 50% of patients classified as stage 2 AKI. Diabetes was the most common comorbidity, followed by cancer, liver disease, cardiovascular disease, and preexisting CKD. Approximately 28% of patients had a previous use of NSAIDs. Sepsis was the most common cause of death, followed by hypovolemia, cardiogenic shock, hepatorenal syndrome, drug-induced AKI, and rhabdomyolysis.^2^ Another study reported that the most common causes of AKI as follows: pre-renal AKI and ischemic acute tubular necrosis (19.6%), contrast-induced nephrotoxicity (15.6%), GN (15.6%), and sepsis-induced AKI (11.7%).^4^ Sepsis was the most important predictor of mortality in most studies.^5^ Kidney biopsy is underutilized for patients with unknown causes of AKI.^6^

## The Diagnosis of AKI in Egypt

Nephrologists in Egypt use serum creatinine and urine output criteria suggested by Kidney Disease Improving Global Outcomes to diagnose AKI.^7^ Newer biomarkers, such as cystatin C and neutrophil gelatinase-associated lipocalin, are used for research purposes only in AKI.

## Nephrology Workforce in Egypt

The nephrology workforce in Egypt is considered the highest among low-income and low- and middle-income African countries, with nearly 21.65 Egyptian nephrologists per million population.^8^ There are three main formal training programs in nephrology in Egypt. The university residency program (3–5 years), the Egyptian fellowship in nephrology program (4 years), and the residency program in Ministry of Health hospitals, university teaching hospitals, and in medical insurance hospitals (3 years). Some private centers may provide informal training to their working nephrologists. The training is different according to the place of training and includes clinical nephrology, RRT, interventional nephrology, and transplantation training. Candidates can train in places where there are no available trainers for kidney transplantation, interventional nephrology, peritoneal dialysis (PD), or clinical nephrology. Hemodialysis training is universal. After training, nephrologists may select to continue practicing in the public sector, private sector, or, most commonly, select both public and private practice.

## Health Care Coverage and Organizational Structure

**Figure 1:**
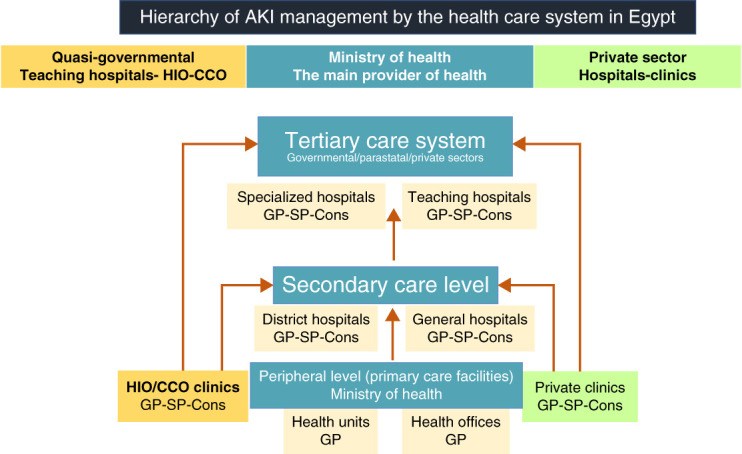
**Hierarchy of AKI management by the healthcare system in Egypt.** CCO, curative care organization; Cons, consultant; GP, general practitioner; HIO, health insurance organization; SP, specialist.

The Egyptian health care system consists of two sectors: public and private. Public health care coverage is either offered through the Ministry of Health or the quasi-governmental insurers represented in the health insurance organization, the curative care organization, and the teaching hospitals/institutes (Figure 1). Patients with AKI may first be seen by a general practitioner, a specialist, or a consultant. Critically ill patients with AKI are referred to tertiary care hospitals with well-developed nephrology-driven services that admit and treat patients with AKI in collaboration with intensivists. Some specialized ICUs are entirely operated by nephrologists who are experienced in the use of bedside ultrasonography to guide fluid management and prescription of RRT.

## RRT

When required, hemodialysis is the main modality of RRT in Egypt. Intermittent hemodialysis is usually available in 100% of tertiary care hospitals, whereas continuous RRT (CRRT) and PD are used less frequently. The use of PD is currently limited because of the high cost of treatment because solutions are being imported from abroad.^9^ CRRT is very costly for most of the patients, is often unavailable, and is not reimbursed. Patients with septic shock on vasopressors with severe AKI are often managed using prolonged intermittent hemodialysis sessions (6–8 hours or more). CRRT is more commonly used in private hospitals. Nephrologists, not intensivists, are responsible for all the medical orders related to the prolonged intermittent hemodialysis or CRRT, including the ultrafiltration rate and the dialysis prescription.

## The Outcomes of AKI in Egypt

For pregnancy-related AKI, outcomes are largely ominous, with maternal mortality ranging from 4% to 22.5%, fetal mortality from 45% to 74%, and progression to ESKD in 4%–37.5% of survivors.^10–12^ Preeclampsia was the most common cause of pregnancy-related AKI, followed by sepsis and peri-partum hemorrhage.^10^ Most patients presented in their third trimester, followed by the puerperium, second trimester, and first trimester.^10,11^ Elderly patients with community-acquired AKI had an in-hospital mortality rate of 16%, and 32% of the elderly survivors developed CKD.^5^ The direct-acting antiviral regimen daclatasvir/sofosbuvir/ribavirin, used for treatment of hepatitis C in Egypt, has been associated with AKI.^13^ Unfortunately, no national data are available on the incidence of hepatitis C virus-induced AKI, especially nowadays after the implementation of the national presidential program to eliminate hepatitis C virus from Egypt.^14^

Regarding hospital-acquired AKI, AKI increases the risk of mortality in patients in the ICU.^2,15^ Elderly patients,^5^ neonates,^16^ and pregnant women^10,11^ had a higher risk of developing AKI during hospitalization. This suggests the need to prioritize treatment of AKI to prevent substantial morbidity and mortality.

## Coronavirus 19 and AKI in Egypt

During the coronavirus disease 2019 (COVID-19) pandemic, AKI was common among hospitalized patients with COVID-19 (14%), with 37% requiring dialysis. The mortality rate in patients with COVID-19 with AKI was 15.4%, which was higher than patients who did not develop AKI (4.8%).^17^

## Challenges and Opportunities

**Figure 2:**
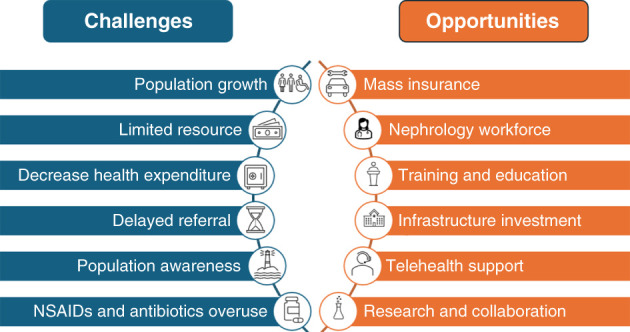
**The challenges of AKI and opportunities to prevent and improve the treatment of AKI in Egypt.** NSAID, nonsteroidal anti-inflammatory drug.

Although Egypt has implemented an extensive basic health service delivery system, the increasing population growth has greatly challenged the public health care system, especially with the continuously declining government expenditure on public health care. Figure 2 summarizes the challenges of AKI and opportunities to prevent and improve the treatment of AKI in Egypt.

In conclusion, this mini-review contributes to the existing body of knowledge on AKI by providing a focused analysis of the Egyptian context. Ultimately, this work may serve as a foundation for future research and public health initiatives aimed at reducing the incidence and improving the management of AKI not only in Egypt but also in similar resource-constrained settings worldwide.^18^
